# Investigating causal mechanisms in randomised controlled trials

**DOI:** 10.1186/s13063-019-3593-z

**Published:** 2019-08-23

**Authors:** Hopin Lee, Robert D. Herbert, Sarah E. Lamb, Anne M. Moseley, James H. McAuley

**Affiliations:** 1 Centre for Statistics in Medicine, Rehabilitation Research in Oxford, Nuffield Department of Orthopaedics Rheumatology and Musculoskeletal Sciences, University of Oxford, Oxford, UK; 2 School of Medicine and Public Health, University of Newcastle, Newcastle, NSW Australia; 3 Neuroscience Research Australia (NeuRA), Sydney, NSW Australia; 4 School of Medical Sciences, University of New South Wales, Sydney, NSW Australia; 5 Institute for Musculoskeletal Health, School of Public Health, University of Sydney, Sydney, NSW Australia; 6 Prince of Wales Clinical School, University of New South Wales, Sydney, NSW Australia

**Keywords:** Mechanism, Mediation analysis, Complex interventions, Causal inference, Musculoskeletal system, Rheumatoid arthritis, Ankle fractures, Exercise therapy

## Abstract

**Introduction:**

In some randomised trials, the primary interest is in the mechanisms by which an intervention exerts its effects on health outcomes. That is, clinicians and policy-makers may be interested in how the intervention works (or why it does not work) through hypothesised causal mechanisms. In this article, we highlight the value of understanding causal mechanisms in randomised trials by applying causal mediation analysis to two randomised trials of complex interventions.

**Main body:**

In the first example, we examine a potential mechanism by which an exercise programme for rheumatoid arthritis of the hand could improve hand function. In the second example, we explore why a rehabilitation programme for ankle fractures failed to improve lower-limb function through hypothesised mechanisms. We outline critical assumptions that are required for making valid causal inferences from these analyses, and provide results of sensitivity analyses that are used to assess the degree to which the estimated causal mediation effects could have been biased by residual confounding.

**Conclusion:**

This paper demonstrates how the application of causal mediation analyses to randomised trials can identify the mechanisms by which complex interventions exert their effects. We discuss methodological issues and assumptions that should be considered when mediation analyses of randomised trials are used to inform clinical practice and policy decisions.

**Electronic supplementary material:**

The online version of this article (10.1186/s13063-019-3593-z) contains supplementary material, which is available to authorized users.

## Introduction

The utility of randomised controlled trials (RCTs) can be extended beyond the estimation of the effects of interventions on health outcomes. In some trials, the interest is in the mechanisms through which the intervention exerts its effects on health outcomes [1]. That is, clinicians and policy-makers may be interested in how the intervention works (or fails to work) through hypothesised causal mechanisms. By identifying the mechanisms of health interventions, researchers and clinicians can refine and adapt interventions to improve the effectiveness of health interventions and guide implementation. This is particularly useful for complex interventions where several mechanisms can be hypothesised to improve health outcomes. The UK Medical Research Council advocates the inclusion of mechanistic analyses in process evaluations of complex interventions [2].

Methods for identifying mediators have been available for decades [3] but recently more rigorous methods for causal mediation analysis have been developed that are based on clearly defined assumptions [4]. To the extent that these assumptions can be met, causal mediation analysis can provide consistent estimates of the extent to which interventions work through particular mechanisms.

In this article, we highlight the value of understanding causal mechanisms in RCTs. We illustrate key concepts by applying causal mediation analyses to two RCTs in physical rehabilitation. In the first example, we examine a potential mechanism by which an exercise programme for rheumatoid arthritis of the hand improves hand function. In the second example, we explore why a rehabilitation programme for ankle fractures failed to improve lower-limb function. We highlight key assumptions and discuss how these analyses may provide clinical and policy implications that extend beyond those provided by standard analyses of RCTs.

### Example of estimating mechanistic effects of an effective intervention (how did the intervention work?)

In the Strengthening And stretching for Rheumatoid Arthritis of the Hand Trial (SARAH) trial (*n* = 490), Lamb et al. [5] showed that an exercise programme for people with rheumatoid arthritis improved hand function more than usual care (total effect = 4.4 [95% confidence interval (CI), 1.5–7.2] points on the 100-point Michigan Hand Outcome Questionnaire at 12 months). After the trial was completed, causal mediation analysis was used to determine how much of the intervention effect was mediated by increases in grip strength. A causal mediation analysis of complete cases (*n* = 387) indicated that 25% of the intervention effect on hand function at 12 months was mediated by increases in grip strength at 4 months (indirect effect = 1.1 [0.3–2.1]). This indicates that exercise improves hand function partly by increasing hand strength. The analysis also suggests that a substantial proportion of the total intervention effect is mediated through other pathways (represented by the direct effect of 3.3 [0.5–6.3]). Future research might seek to identify these alterative mechanisms with the aim of further refining the intervention.

### Example of estimating mechanistic effects of an ineffective intervention (why didn't the intervention have a meaningful effect on the primary outcome?)

In the EXercise or Advice after ankle fracture trial (EXACT) trial (*n* = 214), Moseley et al. [6] tested whether a rehabilitation programme with advice was more effective at improving lower-limb function than advice alone for patients with ankle fracture. A hypothesised mechanism was that rehabilitation would increase physical activity levels, which in turn would improve lower-limb function. In contrast to the SARAH trial, the primary analysis of the EXACT trial did not find evidence of an intervention effect on the primary outcome (total effect = − 0.5 [− 5.0–3.8] on the 80-point Lower Extremity Functional Scale at 3 months). Here the question of interest is why the intervention did not have a meaningful effect on the primary outcome. A causal mediation analysis of complete cases (*n* = 156) found no evidence that physical activity at 1 month influenced function at 3 months (indirect effect = − 0.4 [− 2.1–1.0]). The analysis suggested that, while a change from low to high physical activity would improve lower-limb function by 8.7 points [2.2–15.3], the rehabilitation programme failed to increase physical activity (ratio of odds of being classified as low physical activity = 0.9 [0.6–1.2]). This indicates that although the EXACT investigators identified an important intervention target (physical activity), the rehabilitation programme failed to cause a sufficient change in this target mechanism. This implies that it might be possible to produce functional gains if physical activity levels could be increased using other interventions. These findings could guide the development of new interventions in this population.

### Methodological considerations

As with any causal modelling, the estimates derived from a causal mediation analysis may not be interpretable as causal effects if the underlying assumptions are not met. In the following sections we outline some important assumptions and discuss them within the contexts of the SARAH and EXACT trials.

#### Exchangeability

A critical requirement for robust inference of indirect and direct effects is to satisfy the exchangeability (no-confounding) assumption for the intervention-mediator, intervention-outcome and mediator-outcome effects [4, 7]. In an RCT, the intervention-mediator and intervention-outcome effects can be assumed to be unconfounded because trial participants are randomised to intervention or control conditions. However, the mediator-outcome effect can be confounded because the mediator is not randomised.

**Fig. 1:**
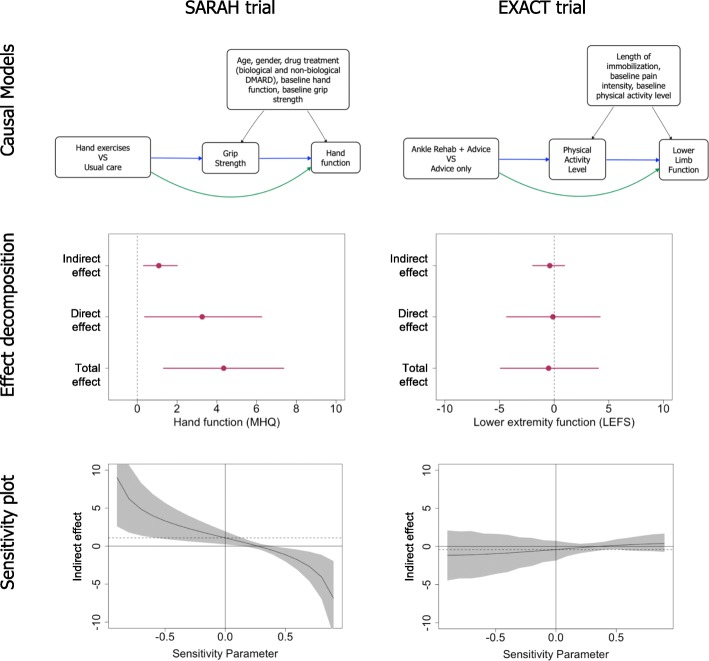
Causal models of intervention mechanisms, effect decomposition, and sensitivity plots of the SARAH and EXACT trials. The causal models panel shows the hypothesised mechanisms of each intervention. The blue lines represent the effect of the intervention on the outcome through the mediator of interest (indirect effect); the green line represents the effect of the intervention on the outcome that is not exerted through the mediator (direct effect) which includes all other possible mechanisms; and the black lines represent possible confounding effects that were adjusted for in the analysis. Each model assumes that the intervention does not modify the mediator-outcome effect. The effect decomposition panel shows how the average total effect of the intervention on the outcome is decomposed into the indirect effect (blue lines in the causal models), and the direct effect (green lines). These effects are presented as unstandardised effects with their 95% confidence intervals. The sensitivity plots show how much the estimated indirect effect would change if there was residual confounding of the mediator-outcome effect. The sensitivity parameter (horizontal axis) represents hypothesised levels of residual confounding: 0 indicates no residual confounding, and − 1.0 and 1.0 are the maximum levels of residual confounding. The dashed horizontal line represents the estimated indirect effect when there is no residual confounding (sensitivity parameter = 0). The curved solid line represents the estimated indirect effect at varied levels of residual confounding. In the SARAH trial, the indirect effect estimate would become 0 if there was moderate residual confounding (sensitivity parameter = 0.30), whereas in the EXACT trial, the indirect effect is stable across levels of residual confounding. The grey zones represent 95% confidence intervals

After adjusting for measured confounders, sensitivity analyses can be used to assess the degree to which the estimated effects could have been biased by residual confounding. For the SARAH trial, sensitivity plots (Fig. 1) show that a moderate level of residual confounding of the mediator-outcome effect would invalidate the indirect effect. This is apparent because the average indirect effect (on the vertical axis) diminishes to 0 when the sensitivity parameter (on the horizontal axis) shifts from 0 (no residual confounding) to 0.30 (moderate residual confounding). In contrast, the sensitivity analysis plots of the EXACT trial shows that the indirect effect would be stable even if there were moderate levels of residual confounding. These sensitivity analyses suggest that the findings of the causal mediation analysis were robust for the EXACT trial but not for the SARAH trial. Researchers conducting causal mediation analyses should always make the no-confounding assumption explicit and report sensitivity analyses. Readers of reports of causal mediation analyses should be aware of the potential impact of residual confounding when interpreting mediation analyses of RCTs.

In some situations, it might also be important to consider interactions between the intervention and confounder(s), and between the mediator(s) and confounder(s), to strongly control for confounding. However, unless there is substantial prior knowledge to believe that exposure-confounder or mediator-confounder interactions will confound the mediator-outcome effect, it may be sufficient to control only for the main effects of the confounders [4]. In SARAH and EXACT, we assumed that there would not be large interaction effects between the interventions and confounders, and between the mediators and confounders. So we only controlled for the main effects of the confounders.

#### The intervention should not have a causal effect on confounders of the mediator-outcome effect

It is important that the intervention is not a cause of confounders of the mediator-outcome effect. In SARAH and EXACT, the confounders were measured before the intervention was delivered, and, therefore, the confounders cannot be caused by the intervention. However, it is possible that there are other unmeasured variables caused by the intervention that could confound the mediator-outcome effect. These variables were not specified in our causal models and therefore did not inform our analysis.

#### Effect modification

In RCTs, it is possible that the effect of a mediator on an outcome could depend on treatment allocation. In other words, allocation may modify the effect of the mediator on the outcome. In the SARAH trial, the effect of grip strength on functional outcomes could depend on whether allocation was to the exercise programme or usual care. Likewise, in the EXACT trial, the effect of physical activity on lower-limb function could depend on whether allocation was to rehabilitation or advice only. When effect modifications are plausible, it is important to account for an intervention-mediator interaction term when specifying the causal model [4]. In practice, including the interaction term will produce indirect and direct effects that are conditional on intervention status. These conditional effects may differ from the average (marginal) effects in the entire population if there is effect modification. In both SARAH and EXACT, the estimated conditional effects were not substantially different from the marginal indirect and direct effects (Additional file 1).

#### Consistency

To make valid causal inferences from mediation analyses, the intervention and mediator must be well-defined [8]. This means that there cannot be multiple versions of the intervention and mediator implemented in the study [9]. In the technical literature, this requirement is often called the consistency assumption [10]. When the consistency assumption is violated and there are multiple versions of the intervention or mediator, interpretation of mediation effects becomes challenging [9].

In the EXACT trial, the challenge is to specifically define what is meant by the proposed mediator, physical activity level; and to articulate the level at which this mediator is fixed when we define our effects of interest. In EXACT, physical activity was classified as ‘low’ or ‘high’ based on metabolic equivalent minutes per week (precise definitions outlined in Additional file 1). In our definition of the indirect effect, we set physical activity to the level taken when individuals are allocated to receive rehabilitation, and the level taken when allocated to advice alone. In our definition of the direct effect, we fixed physical activity to the level taken when allocated to receive rehabilitation. We must assume that all possible versions of the intervention that could set physical activity to a given level will have the same effect on the outcome, lower-limb function. This assumption is at best only approximately satisfied in the EXACT trial.

In the SARAH trial, the consistency assumption concerns the grip-strength mediator. We must assume that, to the extent that there are multiple ways of bringing about change in grip strength, they all have the same effects on lower-limb function. Vanderweele (2012) explains that when consistency is violated and there are multiple versions of the mediator, the indirect effect is likely to be underestimated and the direct effect is likely to be overestimated [9].

## Conclusion

Causal mediation analyses can identify the mechanisms by which health interventions exert their effects. This information can be used to emphasise and refine components of the intervention that operate through effective mechanisms and discard components of the intervention that target ineffective mechanisms. Clinicians and policy-makers could use the findings from causal mediation analyses of RCTs to prioritise critical aspects of multi-component interventions during implementation. Finally, causal mediation analyses can identify which mechanisms failed when promising interventions are found to be ineffective.

## Additional file

Additional file 1: Indirect and direct effects that are conditional on intervention status. Physical activity level cutpoints. Annotated bibliography. (DOCX 338 kb)

## Data Availability

The datasets used and/or analysed during the current study are available from the corresponding author on reasonable request.

## Abbreviations

EXACT: EXercise or Advice after ankle fracture trial
RCT: Randomised controlled trial
SARAH: Strengthening And stretching for Rheumatoid Arthritis of the Hand Trial
