# Supplementary material for: Investigating causal mechanisms in randomised controlled trials
Source: Trials. 2019 Aug 23;20:524. doi: 10.1186/s13063-019-3593-z (PMC6708183; doi:10.1186/s13063-019-3593-z)
Supplement: Supplementary file 1 — Indirect and direct effects that are conditional on intervention status. Physical activity level cutpoints. Annotated bibliography. (DOCX 338 kb) [file 13063_2019_3593_MOESM1_ESM.docx]

**Additional file**

**A1. Indirect and direct effects that are conditional on intervention status**

When an intervention-mediator interaction term is included in the outcome regression model, two conditional effects are produced for the indirect effect and the average direct effect. The solid dot and lines correspond to effects conditional to the intervention group, and the hollow dot and dotted lines correspond to effects conditional to the control group.


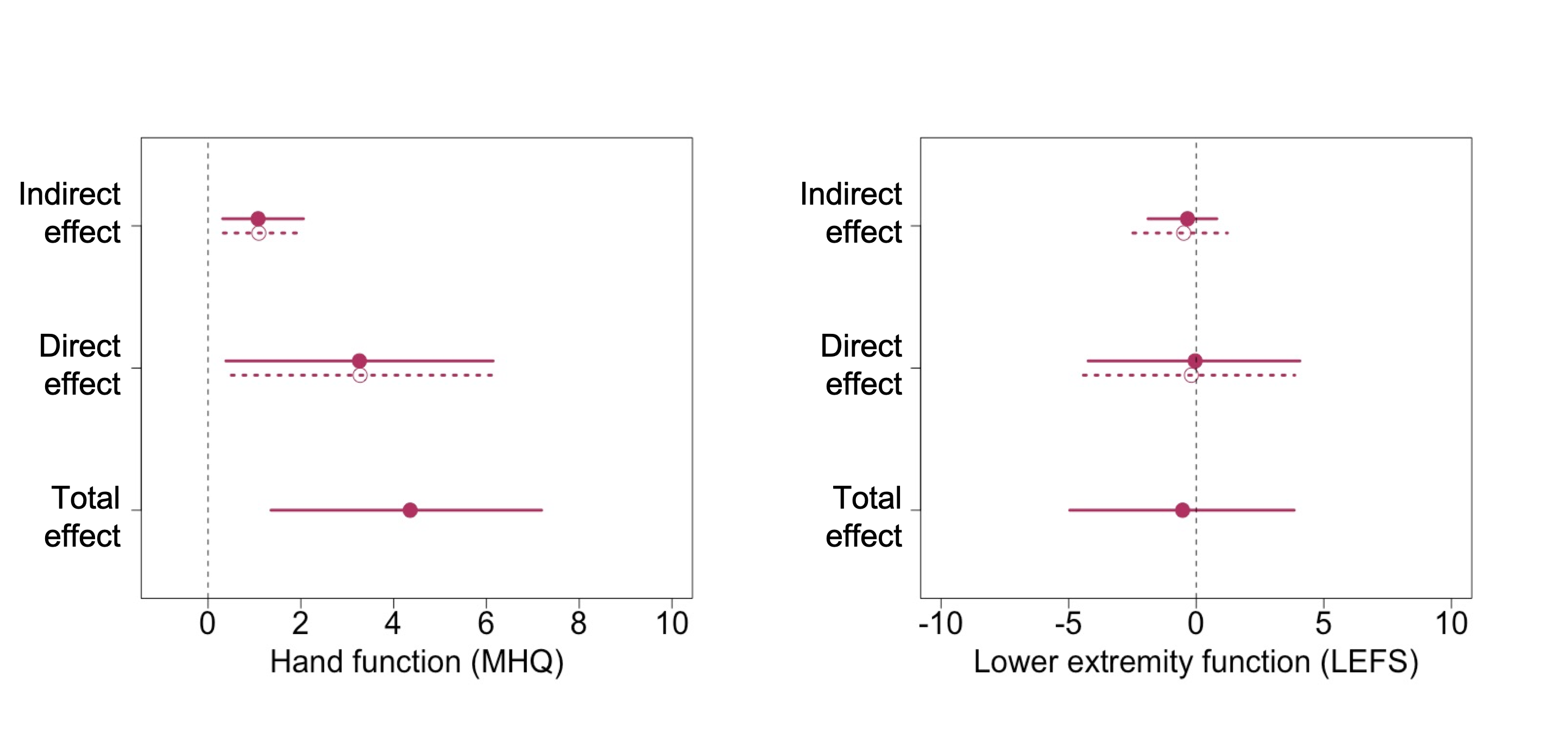


**A2. Physical activity level cutpoints**

High cutpoints: 3 or more days of vigorous-intensity activity achieving 1500MET min/wk or more or 7 days of walking, moderate-intensity, or vigorous-intensity activities achieving 3000METmin/wk or more. Moderate cutpoints: 3 or more days of 20 minutes or more vigorous-intensity activity, or 5 or more days of 30 minutes or more moderate-intensity activity or walking, or 5 or more days of walking, moderate-intensity, or vigorous-intensity activities achieving 600MET min/wk or more. Low cutpoint: not “moderate” or “high”

**A3. Annotated bibliography**

Vanderweele TJ. *Explanation in Causal Inference: Methods for Mediation and Interaction*. Oxford: Oxford University Press; 2015.

A comprehensive textbook that covers theory, methodology, and selected topics on mediation and interaction.

Tingley D, Yamamoto T, Hirose K, Keele L, Imai K. mediation: R Package for Causal Mediation Analysis. *J Stat Softw*. 2014;59(5):1-38.

The analysis in our paper uses the “mediation” R package. Tingley et al. provide comprehensive and userfriendly instructions for implementing the package to conduct causal mediation anlayses and sensitivity analyses. Our implemention of this package to the SARAH and EXACT trial is shared on: <https://github.com/hopinlee/causal-mediation-analysis-of-EXACT-and-SARAH-trials.git>
